# Going off to Oyster College: stress as larvae impacts performance as outplanted adults

**DOI:** 10.1093/conphys/coag027

**Published:** 2026-04-18

**Authors:** Emma Guerrini Romano

**Affiliations:** Department of Biology, University of Washington, Seattle, WA 98195, USA

Oyster-gardening does not require tilling soil or scarecrows but can be trickier than growing tasty tomatoes. Restoration of oyster reefs is a focus of coastal projects due to the slew of benefits they provide to the ecosystem, like filtering water and providing shelter for organisms. To gain these benefits back from degradation, restoration groups raise oysters in controlled conditions to then outplant them to a site of interest.

But unlike our home gardens, oyster reefs experience massive fluctuations in factors like salinity due to their estuarine environment. While critters like crabs and fish can crawl or swim away from these stressful areas, oysters are stuck in place. This means that oysters need to effectively respond to environmental fluctuations to survive. For restoration projects that hope to recover ecosystem services, efforts need to ensure that outplanted oysters have the best chance at surviving in the wild. This begs the question: how do you grow a tough oyster?

That is exactly what a team of researchers at Florida State University (Fuqua and Brooke, 2025) set out to answer. They were interested in the carryover effect, which is the idea that the environment an organism experiences early on might determine how well it copes with stressors later in life. The hope was that exposing farmed oysters to stress as larvae would increase their likelihood of surviving in the wild.

To test this, the team raised Eastern oyster larvae in multiple controlled salinity treatments (Fig. 1). They tracked how many larvae survived and how many reached the competent stage, or the point at which larvae develop the ability to settle. After this stage, they measured juvenile growth. The newly settled young oysters were then transplanted into the real world, specifically Oyster Bay (OB) or Alligator Harbour (AH). I know which site I’d prefer if I were an oyster! Once outplanted, oxygen consumption and condition index (a measure of how well-fed the oyster is) were recorded after several months.

**Figure 1:**
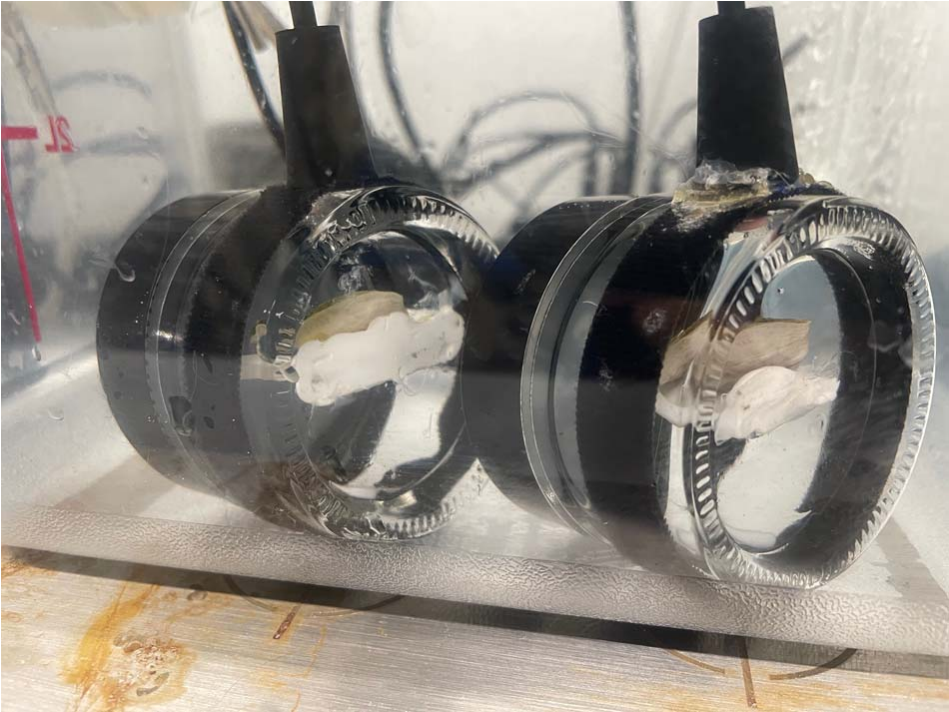
Laboratory grown Eastern oysters. Image credit: Emily Fuqua.

So how did the oysters fare? During the larval stage, low salinity treatments caused slower growth and lower chances of reaching competency. Above 15 ppt until 20 ppt, both parameters peaked. Above 20 ppt, they declined again. But as early juveniles, the trend flipped: oysters raised in low salinity grew about twice as fast as those from high salinity treatments.

The real interest for restoration, though, is what happens once the oysters are out in the wild. And here, where the oysters were planted, made a big difference. Oysters at AH grew faster overall, while those at OB tended to stay smaller but carried more stored energy, shown by their higher condition index. That extra energy could help them weather tough spells. Greater size early on makes AH oysters less likely to be eaten by size-selective predators like blue crabs and oyster drills.

But their early-life salinity exposure still mattered, just differently at each site. At OB, oysters that had been raised in low salinity larval conditions were still feeling the effects months later: they had higher energy use and lower energy reserves than oysters that came from higher salinity larval treatments. At AH, though, those differences mostly disappeared, and oysters performed similarly regardless of how salty their larval environment had been.

Together, the results show that the field site sets the stage, while larval salinity shapes how each oyster performs once it gets there.

## References

[ref1] Fuqua E, Brooke S (2025) Living with the past: larval eastern oyster (*Crassostrea virginica*) culture salinity affects post-metamorphic physiological performance. Conserv Physiol 13: coaf077. 10.1093/conphys/coaf077. 41216001 PMC12596496

